
JOURNAL INFORMATION
==============================
NLM Title Abbreviation: Orthop Surg
Journal ID: Orthop Surg
NLM Journal ID: 101501666
Journal ID: OS

Orthopaedic Surgery

ISSN: 1757-7853
EISSN: 1757-7861
Publisher: Wiley

ARTICLE INFORMATION
==============================
PMCID: PMC11474576
DOI: 10.1111/os.13783
Article ID: OS13783
Article version: 1
Subjects: Issue Information, Issue Information

Issue Information

Electronic publication date: 2024 Mar 10
Publication date: 2024 Mar
Volume: 16
Issue: 3
First page: 521
Last page: 522
Copyright: © 2024 The Authors. Orthopaedic Surgery published by Tianjin Hospital and John Wiley & Sons Australia, Ltd.
License: This is an open access article under the terms of the http://creativecommons.org/licenses/by/4.0/ License, which permits use, distribution and reproduction in any medium, provided the original work is properly cited.
License URL: https://creativecommons.org/licenses/by/4.0/

Figure count: 0
Table count: 0
Page count: 6

source-schema-version-number: 2.0
cover-date: March 2024
details-of-publishers-convertor: Converter:WILEY_ML3GV2_TO_JATSPMC version:6.4.9 mode:remove_FC converted:15.10.2024

==============================

Volume 16 • Issue 3 • March 2024 • ISSN 1757-7861

### Orthopaedic Surgery

Open Access

REVIEW ARTICLES

523
AUTOLOGOUS COSTAL CHONDRAL/OSTEOCHONDRAL TRANSPLANTATION AND COSTA-DERIVED CHONDROCYTE IMPLANTATION FOR ARTICULAR CARTILAGE REPAIR: BASIC SCIENCE AND CLINICAL APPLICATIONS

532
EMERGING ROLES OF MACROPHAGE POLARIZATION IN OSTEOARTHRITIS: MECHANISMS AND THERAPEUTIC STRATEGIES

CLINICAL ARTICLES

551
HOW DOES THE SLUMP SITTING RADIOGRAPH INCREASE PROPORTION OF SEGMENTAL INSTABILITY AND KYPHOTIC ALIGNMENT OF LUMBAR DEGENERATIVE SPONDYLOLISTHESIS?

559
EARLY OUTCOMES OF BONE AUTOGRAFTING IN THE BILATERAL ATLANTOAXIAL JOINTS APPLIED IN POSTERIOR FUSION SURGERY

568
A SELF-DESIGNED ENDOBUTTON INSTALLATION DEVICE FOR CORACOCLAVICULAR STABILIZATION IN ACUTE ROCKWOOD TYPE III ACROMIOCLAVICULAR JOINT DISLOCATION

577
APPLICATION OF THE NEW IRRIGATION PROTOCOL TO REDUCE RECURRENCE RATE IN THE MANAGEMENT OF PERIPROSTHETIC JOINT INFECTION

585
FATTY INFILTRATION OF MULTIFIDUS MUSCLES: AN EASILY OVERLOOKED RISK FACTOR FOR THE SEVERITY OF OSTEOPOROTIC VERTEBRAL FRACTURES

594
THE COMPARISON OF POSTERIOR INTERVERTEBRAL RELEASE COMBINED WITH POSTERIOR COLUMN OSTEOTOMY AND POSTERIOR COLUMN OSTEOTOMY ALONE FOR THE TREATMENT OF MODERATE-TO-SEVERE RIGID SCOLIOSIS: A PROSPECTIVE CONTROLLED STUDY

604
HIP ARTHROSCOPY DEBRIDEMENT COMBINED WITH MULTIPLE SMALL-DIAMETER FAN-SHAPED LOW-SPEED DRILLING DECOMPRESSION IN THE TREATMENT OF EARLY AND MIDDLE STAGE OSTEONECROSIS OF THE FEMORAL HEAD: 14 YEARS FOLLOW-UP

613
COMPARISON OF EN BLOC RESECTION AND INTRALESIONAL EXCISION FOR RE-RESECTION OF GIANT CELL TUMORS OF THE SPINE

620
TREATMENT OF L5-S1 FLOATING CALCIFIED LUMBAR DISC HERNIATION WITH PERCUTANEOUS ENDOSCOPIC INTERLAMINAR DISCECTOMY

628
OSTEOTOMY CORRECTION ANGLE CUT-OFF POINTS CAN GUIDE THE OPERATION TO PREVENT A SIGNIFICANT DECREASE IN PATELLA HEIGHT

637
SIX-PART CLASSIFICATION OF FEMORAL INTERTROCHANTERIC FRACTURES: A CLASSIFICATION METHOD TO IMPROVE THE DIAGNOSIS RATE OF UNSTABLE FRACTURES

654
EFFECT OF KNEE VALGUS DEFORMITY ON SYMPTOMATIC VENOUS THROMBOEMBOLISM AND PROSTHESIS REVISION RISK AFTER TOTAL KNEE ARTHROPLASTY: A MULTICENTER RETROSPECTIVE STUDY

662
TIROBOT-ASSISTED PERCUTANEOUS CANNULATED SCREW FIXATION FOR ELDERLY PATIENTS WITH FRAGILITY FRACTURES OF THE PELVIS: A RETROSPECTIVE STUDY

RESEARCH ARTICLES

675
HIGH-SPEED CENTRIFUGATION EFFICIENTLY REMOVES IMMUNOGENIC ELEMENTS IN OSTEOCHONDRAL ALLOGRAFTS

687
BIOMECHANICAL COMPARISON OF ANTERIOR CERVICAL CORPECTOMY DECOMPRESSION AND FUSION, ANTERIOR CERVICAL DISCECTOMY AND FUSION, AND ANTERIOR CONTROLLABLE ANTEDISPLACEMENT AND FUSION IN THE SURGICAL TREATMENT OF MULTILEVEL CERVICAL SPONDYLOTIC MYELOPATHY: A FINITE ELEMENT ANALYSIS

700
ANALYSIS OF THE POTENTIAL ANGIOGENIC MECHANISMS OF BUSHENHUOXUE DECOCTION AGAINST OSTEONECROSIS OF THE FEMORAL HEAD BASED ON NETWORK PHARMACOLOGY AND EXPERIMENTAL VALIDATION

718
BLEEDER'S FEMUR: THE PROXIMAL FEMORAL MORPHOLOGY IN HEMOPHILIC PATIENTS WHO UNDERWENT TOTAL HIP ARTHROPLASTY

724
PARAVERTEBRAL MUSCULAR NEUROPHYSIOLOGICAL FUNCTION AS AN INDEPENDENT OUTCOME PREDICTOR OF RECURRING HERNIATION/LOW BACK PAIN AFTER RADIOFREQUENCY ABLATION: A PROSPECTIVE FOLLOW-UP AND CASE-CONTROL STUDY BASED ON SURFACE ELECTROMYOGRAPHY

733
DEXAMETHASONE INDUCED OSTEOCYTE APOPTOSIS IN STEROID-INDUCED FEMORAL HEAD OSTEONECROSIS THROUGH ROS-MEDIATED OXIDATIVE STRESS

OPERATIVE TECHNIQUES

745
DYNAMIC ANTERIOR STABILIZATION WITH HILL-SACHS REMPLISSAGE CAN BE EMPLOYED IN SKELETALLY IMMATURE PATIENTS—OPERATIVE TECHNIQUE

754
ANTERIOR FULL-ENDOSCOPIC SINGLE-PORT DOUBLE TRANSCORPOREAL SPINAL CORD DECOMPRESSION FOR NONCONTINUOUS TWO-SEGMENT CERVICAL SPONDYLOTIC MYELOPATHY: A TECHNICAL NOTE

766
SUBTROCHANTERIC OSTEOTOMY IN DIRECT ANTERIOR APPROACH TOTAL HIP ARTHROPLASTY FOR CROWE IV DYSPLASIA—SURGICAL TECHNIQUE AND LITERATURE REVIEW

CASE REPORTS

775
ARTHROSCOPIC LINEAR CHAIN FIXATION FOR THE TREATMENT OF MEDIAL MENISCUS POSTERIOR ROOT TEAR: A CASE STUDY

781
PAY ATTENTION TO THE OSTEOCHONDROMAS IN FIBRODYSPLASIA OSSIFICANS PROGRESSIVA

WILEY

**View this journal online at wileyonlinelibrary.com/journal/os**

### Orthopaedic Surgery

**Honorary Editor-in-Chief**
Guixing Qiu, *Beijing, China*

**Editor-in-Chief**
Xinlong Ma, *Tianjin, China*

**Associate Editors**
Cyrus Cooper, *Southampton, UK*
Benjamin G. Domb, *Des Plaines, USA*
Qing Jiang, *Nanjing, China*
Baoge Liu, *Beijing, China*
Zhuojing Luo, *Xi'an, China*
Luigi Meccariello, *Moiano, Italy*
Ling Qin, *Hong Kong, China*
Qi Yin, *Liverpool, UK*

**Editorial Board**
Elisa Belluzzi, *Padua, Italy*
Carlo Biz, *Padua, Italy*
Mary L Bouxsein, *Boston, USA*
Richard E. Buckley, *Calgary, Canada*
Zhengdong Cai, *Shanghai, China*
Wei Chai, *Beijing, China*
Bohua Chen, *Qingdao, China*
Liang Chen, *Soochow, China*
Yanxi Chen, *Shanghai, China*
Pramod Devkota, *Patan, Nepal*
Meng Fan, *Tianjin, China*
Haoyu Feng, *Taiyuan, China*
Maoqi Gong, *Beijing, China*
Yong Hai, *Beijing, China*
Wei Huang, *Chongqing, China*
Baochao Ji, *Urumqi, China*
Ayhan Kanat, *Rize, Turkey*
Kenny Kwan, *Hong Kong, China*
Sallie Lamb, *Exeter, UK*
Lifeng Lao, *Shanghai, China*
Jung-Hee Lee, *Seoul, Korea*
Guanghua Lei, *Changsha, China*
Guoan Li, *Newton, USA*
Hui Li, *Tianjin, China*
Jianmin Li, *Jinan, China*
Weishi Li, *Beijing, China*
Jianhao Lin, *Beijing, China*
Fanxiao Liu, *Munich, Germany*
Ruben Lostado, *La Rioja, Spain*
Songchen Lyu, *Harbin, China*
Weihu Ma, *Ningbo, China*
Xin Ma, *Shanghai, China*
Masaya Nakamura, *Tokyo, Japan*
Muthu Sathish, *Velayuthampalayam, India*
Wolfgang Senker, *Linz, Austria*
Sancar Serbest, *Boston, USA*
Cailiang Shen, *Hefei, China*
Bin Shen, *Chengdu, China*
Huiyong Shen, *Shenzhen, China*
Jianxiong Shen, *Beijing, China*
Puyi Sheng, *Guangzhou, China*
Junying Sun, *Soochow, China*
Shui Sun, *Jinan, China*
YasuhikoTakegami, *Nagoya, Japan*
Sakae Tanaka, *Tokyo, Japan*
Xiaodong Tang, *Beijing, China*
Fei Wang, *Shijiazhuang, China*
Weijun Wang, *Nanjing, China*
Xu Wang, *Shanghai, China*
Zheng Wang, *Beijing, China*
Jidapa Wongcharoenwatana, *Bangkok, Thailand*
Chi-Chuan Wu, *Taipei, China*
Nan Wu, *Beijing, China*
Zhihong Wu, *Beijing, China*
Yayi Xia, *Lanzhou, China*
Jianru Xiao, *Shanghai, China*
Zhongyu Xie, *Shenzhen, China*
Dan Xing, *Beijing, China*
Weiguo Xu, *Tianjin, China*
Peng Xu, *Xi 'an, China*
CaoYang, *Wuhan, China*
Pei Yang, *Xi 'an, China*
Zhaoming Ye, *Hangzhou, China*
Xiuchun Yu, *Jinan, China*
Bin Yu, *Guangzhou, China*
Yi Zeng, *Chengdu, China*
Dianying Zhang, *Beijing, China*
Hong Zhang, *Texas, USA*
Weibin Zhang, *Shanghai, China*
Xianlong Zhang, *Shanghai, China*
Jianguo Zhang, *Beijing, China*
Bin Zhao, *Taiyuan, China*
Jie Zhao, *Shanghai, China*
Yu Zhao, *Beijing, China*
Zongke Zhou, *Chengdu, China*
Yue Zhu, *Shenyang, China*
Zezhang Zhu, *Nanjing, China*

**Address for Editorial Correspondence:** Editorial Board of *Orthopaedic Surgery*, Tianjin Hospital, 122 Munan Road, Tianjin 300050, China (email: orthopaedicsurgery@126.com; telephone: +86 22 2833 4734; fax: +86 22 2824 1184).

**Disclaimer:** The Publisher, Tianjin Hospital and Editors cannot be held responsible for errors or any consequences arising from the use of information contained in this journal; the views and opinions expressed do not necessarily reflect those of the Publisher, Tianjin Hospital and Editors, neither does the publication of advertisements constitute any endorsement by the Publisher, Tianjin Hospital and Editors of the products advertised.

**Copyright © 2024 The Authors. Orthopaedic Surgery published by Tianjin Hospital and John Wiley & Sons Australia, Ltd**

**Copyright and Copying (in any format)**
Copyright © 2024 The Authors. Orthopaedic Surgery published by Tianjin Hospital and John Wiley & Sons Australia, Ltd. All rights reserved. No part of this publication may be reproduced, stored or transmitted in any form or by any means without the prior permission in writing from the copyright holder. Authorization to copy items for internal and personal use is granted by the copyright holder for libraries and other users registered with their local Reproduction Rights Organisation (RRO), e.g. Copyright Clearance Center (CCC), 222 Rosewood Drive, Danvers, MA 01923, USA (www.copyright.com), provided the appropriate fee is paid directly to the RRO. This consent does not extend to other kinds of copying such as copying for general distribution, for advertising or promotional purposes, for republication, for creating new collective works or for resale. Permissions for such reuse can be obtained using the RightsLink "Request Permissions" link on Wiley Online Library. Special requests should be addressed to: permissions@wiley.com

For submission instructions, subscription and all other information visit: http://wileyonlinelibrary.com/journal/os

ISSN 1757-7861 (Online)

OS.JEB.March24
